# The role and mechanism of dapagliflozin in Alzheimer disease: A review

**DOI:** 10.1097/MD.0000000000039687

**Published:** 2024-09-27

**Authors:** Ping Chen, Lihui Liang, Yuhan Dai, Shan Hui

**Affiliations:** a Department of Geriatrics, Hunan Provincial People’s Hospital Hunan Normal University First Affiliated Hospital, Changsha, China.

**Keywords:** Alzheimer disease, dapagliflozin, interview, mechanism, SGLT2 inhibitors.

## Abstract

Alzheimer disease (AD), as the main type of dementia, is primarily characterized by cognitive dysfunction across multiple domains. Current drugs for AD have not achieved the desired clinical efficacy due to potential risks, inapplicability, high costs, significant side effects, and poor patient compliance. However, recent findings offer new hope by suggesting that sodium-glucose cotransporter 2 inhibitors (SGLT-2i) may possess neuroprotective properties, potentially opening up novel avenues for the treatment of AD. This review delves deeply into the multifaceted mechanisms of action of SGLT-2i in AD, encompassing antioxidative stress, antineuroinflammation, upregulation of autophagy, antiapoptosis, acetylcholinesterase inhibitor activity, and protection of endothelial cells against atherosclerosis and damage to the blood-brain barrier, among others. Furthermore, it provides an overview of recent advances in clinical research on this drug. These findings suggest that SGLT-2i is poised to emerge as a pivotal candidate for the treatment of AD, given its diverse functional effects.

## 1. Introduction

With the global population aging, it is anticipated that the number of individuals suffering from dementia will sharply increase to 113 million by 2050, nearly 2.5× higher than in 2010.^[1]^ Of particular concern is the notable rise in dementia cases in low-education, low-income, and middle-developed countries.^[2–4]^ According to statistics, global spending on Alzheimer disease (AD) has reached a staggering $305 billion by 2020 and is projected to exceed $1 trillion by 2050.^[5]^

Currently, despite the emergence of therapies based on AD pathophysiology, such as gene therapy, mitochondrial immune targeting, and nano-assisted technologies, these therapies face challenges in achieving significant results in clinical practice due to their limited applicability, potential risks, and high costs.^[6,7]^ Although monoclonal antibody therapy against amyloid-like proteins has shown some promise in phase III clinical trials, its specific effects and long-term outcomes remain uncertain.^[8]^

Existing FDA-approved drugs, such as acetylcholinesterase (AChE) inhibitors (donepezil and galantamine) and the N-methyl-D-aspartate receptor antagonist memantine, have shown efficacy in the treatment of mild-to-moderate cognitive impairment. However, due to the presence of side effects, patient adherence is suboptimal. Nevertheless, recent advancements in research have shed new light on the management of AD. Studies suggest that sodium-glucose cotransporter 2 inhibitors (SGLT-2i) may have neuroprotective properties, opening up new possible directions for the treatment of AD.^[9]^ Specifically, a retrospective study revealed that patients with type 2 diabetes who were prescribed SGLT-2i had a relatively low risk of dementia, with the lowest risk observed among those taking dapagliflozin (DAPA).^[10]^

Therefore, this paper will further explore the role and mechanism of DAPA in AD, including its antineuronal inflammation, antioxidative stress, upregulation of autophagy, antiapoptosis effects, cholinesterase inhibition, and protection of endothelial cells against atherosclerosis (AS) and maintenance of blood-brain barrier (BBB) integrity.

## 2. The etiology, clinical manifestations, and pathophysiology of AD

AD is a significant factor in dementia and all-cause mortality. The etiology of AD encompasses both hereditary and nonhereditary factors. Hereditary risk factors can be categorized into familial and sporadic types. Familial factors are primarily characterized by mutations in specific genes located on chromosomes, such as amyloid precursor protein (APP) on chromosome 21, presenilin 1 on chromosome 14, and presenilin 2 on chromosome 1^[11,12]^; the onset occurs before the age of 65 years.^[13]^ Sporadic AD accounts for approximately 90% of patients and is closely associated with carrying the Apolipoprotein E4 allele, which generally occurs well after the age of 65 years.^[14]^ Nongenetic high-risk factors for AD include increased age and metabolic abnormalities such as diabetes, hyperlipidemia, hypertension, and obesity, as well as hearing impairment, alcohol abuse, and traumatic brain injury.^[3,15]^

The clinical presentation of AD can range from mild cognitive decline in the early stages or solely psychological symptoms such as anxiety, depression, delusions, and hallucinations to severe impairments across multiple cognitive domains that significantly impact the quality of life in later stages. These impairments include memory loss, impaired language function, diminished spatial orientation, and reduced executive ability.^[3,16]^

**Figure 1.**
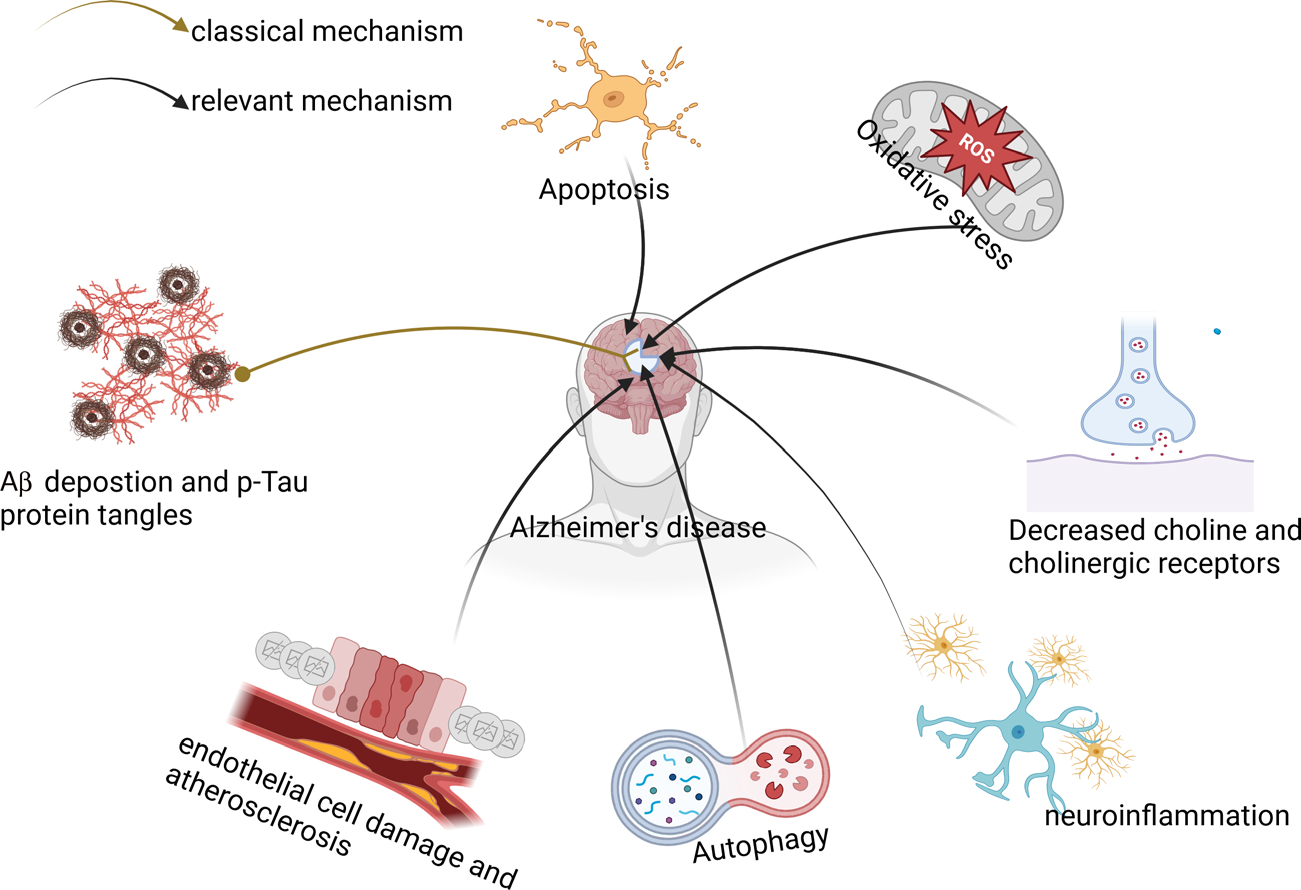
The pathogenesis of Alzheimer disease. Aβ = amyloid-beta.

At the pathological level, AD is characterized by the deposition of extracellular amyloid-beta (Aβ) and the formation of intracellular tangles of neuronal fibers composed of hyperphosphorylated tau proteins that are highly coiled within neurons.^[17,18]^ The plaques formed by Aβ deposits and hyperphosphorylated tau protein disrupt the normal function of neurons and synapses, representing the classic pathogenesis of AD.^[19,20]^ Moreover, the pathogenesis of AD also involves complex processes such as oxidative stress, neuroinflammation, dysautophagy, and neuronal apoptosis (Fig. 1).

In summary, AD is a multifactorial and mechanistically complex disorder involving genetic and environmental interactions. In-depth research and understanding of these factors and mechanisms are crucial for the prevention, early diagnosis, and effective treatment of AD.

## 3. Dapagliflozin

The SGLT-2i DAPA was initially approved for the reduction of blood glucose levels. However, further research has revealed its significant medical benefits, including a reduced risk of cardiovascular death and heart failure events,^[21]^ as well as effective reduction in urinary protein excretion and delayed progression of renal failure.^[22]^ In addition, it exhibits antifibrotic effects on multiple organs,^[23]^ opening up new possibilities for the treatment of various diseases.

**Figure 2.**
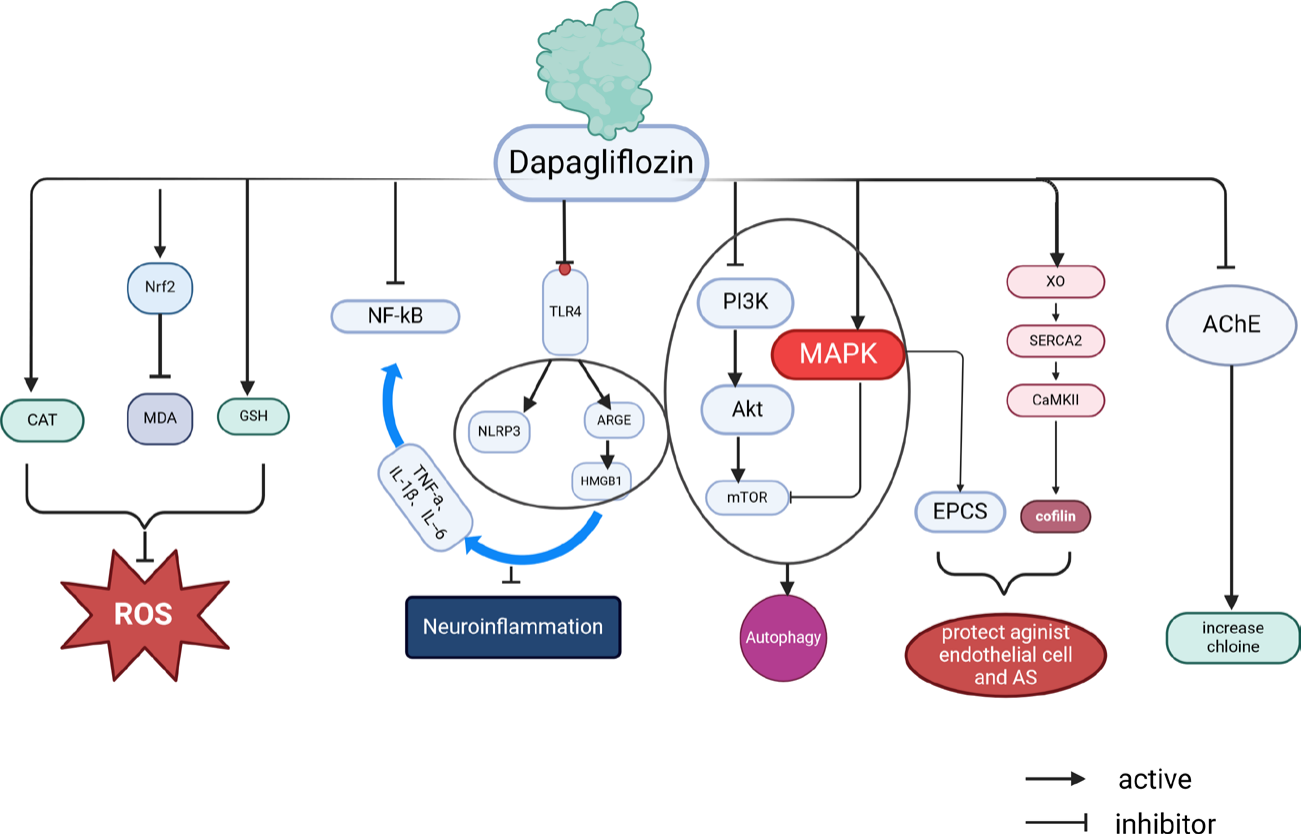
Mechanism of action of dapagliflozin in the treatment of Alzheimer disease. AChE = acetylcholinesterase, Akt = protein kinase, ARGE = Receptor for advanced glycosylation end products, AS = atherosclerosis, CaMKII = calmodulin-dependent kinase II, CAT = Catalase, EPC = endothelial progenitor cell, GSH = glutathione, HMGB1 = high-mobility group protein box 1, IL = Interleukin, MAPK = Adenylate-activated protein kinase, MDA = malondialdehyde, mTOR = Mammalian target of rapamycin, NF-κB = nuclear transcription factor, NLRP3 = nucleotide-binding oligomerization domain-like receptor protein 3, Nrf2 = nuclear factor erythroid 2-related factor, PI3K = phosphatidylinositol 3-kinase, ROS = reactive oxygen species, SERCA2 = sarcoplasmic reticulum Ca2+-ATPase, TLR4 = toll-like receptor 4, TNF-α = tumour necrosis factor-α, XO = xanthine oxidase.

Recent studies have demonstrated the presence of sodium-glucose cotransporters (SGLTs) in the mammalian nervous system, with SGLT2 being highly expressed in the hippocampus, cerebellum, and BBB endothelial cells.^[24–28]^ This specific distribution pattern provides compelling scientific evidence for the protective role of DAPA in the nervous system.^[9]^ Significantly, a recent large cohort study revealed that DAPA reduced the risk of dementia,^[29]^ including one of its leading causes: AD. Based on these research findings, this article will focus on exploring the potential role and mechanism of DAPA in AD, aiming to offer new insights and approaches for treating this neurodegenerative disease(Fig. 2).

## 4. Other mechanisms of AD and the role of DAPA

### 4.1. The interaction between oxidative stress and AD

In AD, the production of toxic, neurotropic Aβ42 peptides can significantly induce oxidative stress responses.^[30,31]^ Oxidative stress impacts the function of NAD-dependent protein deacetylase, leading to mitochondrial dysfunction and affecting energy metabolism.^[32–34]^ The overproduction of oxidative stress products not only leads to oxidative modification of glycoproteases, triggering abnormal glucose metabolism, and reduced ATP synthesis,^[35,36]^ but also the potential generation of neurotoxic late glycan terminal products.^[37]^ In addition, this stimulation can trigger dysfunction within the ubiquitin-protease system, causing abnormal aggregation of proteins inside and outside cells.^[38]^

Reduced ATP synthesis diminishes the efficiency of action potential generation and neural signal transmission, compromising the ability of neurons to maintain ion gradient balance and leading to intracellular Ca2+ overload, which promotes synapse and neuronal death.^[36,39]^ Mitochondrial dysfunction triggers the release of cytochrome C and apoptosis-inducing factors, further exacerbating neuronal death.^[36,40,41]^ Abnormal protein accumulation can also induce oxidative stress. The above ultimately results in a vicious cycle that causes persistent damage to nerve cells and synapses, worsening the clinical symptoms of AD.

#### 4.1.1. Mechanism of oxidative stress against DAPA

In neurodegenerative diseases, the expression level of oxidative stress products is inversely proportional to the level of the nuclear factor erythroid 2-related factor (Nrf2) but directly proportional to the severity of the disease.^[42]^ Recent studies have demonstrated that in AD model rats treated with lipopolysaccharide, there was a 73.17% decrease in the oxidative stress product malondialdehyde and a 67.57% decrease in Nrf2 content compared to control rats. However, when rats were administered DAPA plus lipopolysaccharide, their total antioxidant capacity increased by 1.22-fold, and their Nrf2 content increased by 98.23%.^[43]^ This discovery suggests that DAPA can activate the Nrf2 signaling pathway(particularly, Silent information regulator 1/Nrf2/Heme oxygenase-1), enhancing antioxidant capacity and effectively combating damage to the central nervous system caused by reactive oxygen species (ROS).^[43,44,45]^ Furthermore, Amin et al^[46]^ also demonstrated that the neuroprotective effect of SGLT2 is achieved through the reduction of oxidative stress. Specifically, SGLT2 decreases malondialdehyde levels, enhances catalase activity, and increases the content of antioxidant glutathione in brain tissue. Therefore, DAPA plays a crucial role in AD treatment by reducing oxidative stress products associated with AD, restoring neuronal energy metabolism, improving neurons’ capacity to maintain ion gradient balance, and safeguarding mitochondrial function.

### 4.2. Correlation between the neuroinflammatory response and AD

AD is closely associated with a neuroinflammatory response.^[47]^ The theoretical basis mainly encompasses the following aspects. First, in patients with AD, there is a significant presence of proinflammatory cytokine IL-1β. This cytokine not only regulates the synthesis of APP but also directly participates in the synthesis of Aβ.^[48]^ Second, excessive deposition of Aβ can activate the classical pathway of complement, starting from the initial activation of complement C1 components to the formation of the Membrane attack complex, which triggers a proinflammatory cascade known as the membrane attack complex.^[49–51]^ In addition, Aβ hyperactivates microglia and astrocytes through immune regulation and maintenance of neuronal homeostasis. This leads to their hyperfunction and accelerates the removal of normal neurons and synaptic connections, further activating the complement system.^[52,53]^ It has also been discovered that the penetration of Aβ into the healthy neural tissue areas of patients with AD can further stimulate microglia to release significant amounts of proinflammatory factors, such as IL-6.^[54]^ Meanwhile, Aβ is capable of generating advanced glycation products by inducing oxidative stress, which indirectly causes neuronal damage and promotes the release of inflammatory mediators, such as IL-1 and tumor necrosis factor-α (TNF-α).^[37]^ These processes collectively exacerbate the inflammatory response in AD.

#### 4.2.1. Mechanism of antineuroinflammatory action of DAPA

Research has demonstrated the presence of toll-like receptor 4 (TLR4) overexpression in patients with AD.^[55]^ TLR4 overexpression enhances the activity of the nucleotide-binding oligomerization domain-like receptor protein 3 (NLRP3) inflammasome and its downstream mediators, leading to diffuse neuritis in the brain.^[56]^ It also activates the expression of high-mobility group protein box 1 (HMGB1) and receptor for advanced glycation end products. HMGB1 interacts with stromal cell-derived factor-1, producing IL-1β, IL-6, and TNF-α in hippocampal neurons, inhibiting microglial phagocytosis, reducing Aβ peptide clearance, and exacerbating AD’s pathological progression.

Studies show that DAPA effectively reduces inflammation by inhibiting TLR4/advanced glycation end products/HMGB1 signaling, TLR4/NLRP3 inflammasome signaling, and nuclear transcription factor activation.^[57–60]^ DAPA's inhibitory effect on these pathways suggests its potential as an important antineuroinflammatory agent in AD treatment.

### 4.3. The interrelationship between autophagy and AD development

Autophagy, a central mechanism for maintaining cell survival and organismal homeostasis, plays a crucial role. Autophagy-related diseases often affect the nervous system, especially when autophagy is dysregulated. Many studies have shown that neurodegenerative diseases, including AD, are often accompanied by abnormal protein aggregation, and the process of autophagy is closely linked to the clearance of these proteins.

However, in these disease states, the function of autophagy is often inhibited.^[10,61]^ The strong correlation between autophagy and AD is evident from the observation of a significantly expanded autophagy compartment in patients with AD.^[62]^ Further analysis of multilayer brain tissue proteins in patients at different stages of AD revealed accumulation of the autophagic substrate Sequestosome 1, indirectly suggesting a reduction in autophagy flux: a finding similar to that observed in an AD laboratory model.^[63]^

#### 4.3.1. DAPA can upregulate autophagy

The autophagic cascade is a complex process involving the initiation and selective recruitment of autophagy-associated proteins.^[64]^ Under physiological conditions, the autophagy cascade is inhibited by rapamycin kinase complex 1 (mTORC1), and the activity of mTORC1 is regulated by adenylate-activated protein kinase (AMPK) and phosphatidylinositol 3-kinase (PI3K)/protein kinase (AKT) pathways. The activation of AMPK during energy imbalance or nutrient deprivation can counteract the inhibitory effect of mTORC1 on autophagy.

Recent studies have shown that DAPA activates AMPK in the hippocampus^[65]^ and suppresses the PI3K/AKT/mTOR pathway.^[43]^ This is consistent with previous suggestions that the antagonistic effect of SGLT-2i on neurological disorders may originate from their modulation of the PI3K/AKT/endothelial nitric oxide synthase signaling pathway, which subsequently affects mTOR activation.^[44]^ mTORC1 catalyzes the phosphorylation of autophagy initiators Unc-51-like kinase 1 and autophagy-associated protein 13, ultimately affecting autophagosome formation.^[66]^ By inhibiting mTORC1, DAPA elevates autophagy and promotes the formation of autophagosomes, thus enhancing the degradation of intracellular or extracellular aggregated proteins (including tau and Aβ deposition in AD). These findings provide new perspectives for DAPA in therapeutic strategies for AD.

### 4.4. Interrelationship between abnormal apoptosis and AD

Apoptosis is an active, programmed cell death process initiated by internal and external factors. It plays a crucial role in biological evolution, the maintenance of environmental homeostasis, and phylogeny. In mammals, apoptosis transmits death signals through exogenous and endogenous pathways that activate caspase-containing enzyme lines. These enzymes execute cell death by upregulating apoptotic signals or activating downstream enzymes to form apoptotic bodies. Eventually, these bodies are cleared by phagocytes or the ubiquitin-protein lysosomal system.^[67–69]^ However, the occurrence of an abnormal apoptotic program may lead to a range of disorders, including neurodegenerative disorders. It has been demonstrated that caspase activation is closely linked to degenerative granule vacuolation in the hippocampus. Specifically, caspase-3 cleaves APP, resulting in an increase in Aβ and hyperphosphorylation of tau, leading to the formation of neurofibrillary tangles and inducing neurotoxicity.^[68,70]^ Furthermore, numerous studies have indicated abnormally elevated expression of apoptotic proteins in patients with AD, along with the detection of activated caspases and their cleavage products, as well as the presence of apoptosis response protein-4 in prostate cells.^[68]^ In addition, apoptotic pathways are also abnormal in patients with AD, characterized by a loss of function of antiapoptotic molecules (e.g., Antiapoptotic protein) and enhanced activity of pro-apoptotic molecules (e.g., Proapoptotic protein and caspase-3).^[71]^

#### 4.4.1. Mechanism of antiapoptosis in DAPA

In patients with AD, apoptosis occurs as a result of multiple factors. Among these, the accumulation of oxidative stress products can lead to intracellular Ca2+ overload, which can damage the mitochondrial membrane, resulting in the release of cytochrome C and apoptotic factors, ultimately leading to neuronal death. In addition, decreased autophagy contributes to the inefficient degradation of intracellular abnormal substances by phagocytes or the ubiquitin-lysosomal system, thereby exacerbating neuronal death. Furthermore, inflammatory factors also play an important role in the process of cell apoptosis.

It is noteworthy that DAPA can reduce oxidative stress-mediated neuronal death by regulating the extracellular regulated protein kinases 1/2/Cytosolic phospholi-pase A2/Atherosclerosis/ROS pathway,^[72]^ can be capable of modulating the expression of the antiapoptotic factor TP53-induced gly-colysis and apoptosis regulator, which is induced by the tumor suppressor gene protein,^[42]^ and can enhance autophagy pathways to reduce neuronal apoptosis.^[44]^ In addition, DAPA can reduce the levels of inflammatory mediators such as TNF-α to resist the development of apoptosis,^[73,74]^ and repress the expression of antiapoptotic MicroRNA-21.^[75]^ These findings suggest that DAPA has potential applications in AD treatment.

### 4.5. Effect of impaired endothelial cells and AS in AD

Late-onset AD in middle-aged and elderly individuals is closely linked to metabolic changes, including hyperglycemia, hyperlipidemia, and obesity.^[76]^ These metabolic changes often result in alterations in vascular structure and function. Postmortem examination of brain tissue from patients with AD has revealed impaired BBB function and significant vascular changes.^[77,78]^ Thomas et al^[79]^ demonstrated that Aβ induces endothelial dysfunction and damage in rat aorta through the production of ROS. Impaired endothelial cells can lead to subendothelial lipid deposition, ultimately contributing to AS. Notably, cerebrovascular AS is a prominent feature of AD.^[80]^ In a large cohort study, Arvanitakis et al^[81]^ found a positive association between the degree of AS and cognitive decline, as well as the risk of developing AD.

#### 4.5.1. DAPA protective effects on vascular endothelial cells and antiatherosclerosis

In patients with AD, persistent neuroinflammatory responses and oxidative stress lead to the destruction of the vascular endothelium. This further exacerbates intracranial vascular AS and damage to the BBB, impacting not only the supply of blood flow to brain tissue but also resulting in neuronal damage, thus worsening the condition of AD.

Numerous studies point to the role of DAPA in protecting endothelial cell function and preventing microvascular damage. For example, it effectively reduces cardiac microvascular damage and endothelial dysfunction during ischemia/reperfusion by inhibiting the xanthine oxidase (XO)-sarcoplasmic reticulum Ca2+-ATPase (SERCA2)-calcium/calmodulin-dependent kinase II (CaMKII)-cofilin pathway.^[82]^ In addition, it can inhibit inflammation and oxidative stress by activating AMPK and restore the vascular production capacity of endothelial progenitor cells.^[83]^ More importantly, DAPA inhibits the cell surface receptor TLR4 expression and the activation of the nuclear transcription factor, thereby reducing the release of proinflammatory mediators and further protecting endothelial cells.^[60]^ Thus, by improving vascular endothelial function, DAPA has the potential to be an effective drug for AD.

### 4.6. The relationship between cholinesterase enzymes and AD development

Acetylcholine is one of the important neurotransmitters in the human brain, primarily located in the forebrain, cerebral cortex, and basal ganglia areas. Its core function is to participate in the electrophysiological activity between neurons, ensuring signal transmission between neurons.^[84]^ In 1976, the cholinergic hypothesis revealed the important role of acetylcholine in AD, emphasizing that synaptic loss and atrophy in AD impair neurotransmitter conduction.^[85]^ Several studies have explored the alteration of cholinergic signaling mainly during early AD in the presynaptic membrane, with the loss of cholinergic neurons in the basal nucleus and cingulate gyrus. As the condition deteriorates, 90% of the basal loss occurs in the nucleus.^[86–88]^

Reduced binding of acetylcholine and cholinergic receptors is one of the important reasons for the emergence of psychiatric symptoms in patients with AD. This is also the main mechanism by which AChE inhibitors (such as donepezil and galantamine) are used to improve cognitive function in patients with mild-to-moderate AD.

#### 4.6.1. DAPA acts as a cholinesterase inhibitor

Given the significant role of choline levels in neurotransmitter transmission, increasing choline levels can potentially alleviate symptoms associated with AD. Notably, a recent Mendelian randomization analysis revealed a causal relationship between SGLT-2i and choline metabolites. Specifically, it was found that SGLT-2i increased the levels of total choline, phosphatidylcholine, and glycine in whole blood.^[89]^ Furthermore, in an additional experimental study conducted by Arafa et al,^[90]^ using a rat model with memory dysfunction induced by scopolamine, it was discovered that SGLT2 inhibition could significantly reduce AChE activity and increase monoamine levels, leading to an improvement in memory dysfunction in mice.

This finding aligns with another study examining the molecular interaction between DAPA and AChE; by docking the catalytic sites of DAPA and AChE, the investigators found that the free binding energy in the domain was −6.28 kcal/mol, suggesting a tight binding that reduces cholinesterase in blood, implying that DAPA may act as a potential AChE inhibitor. Similar conclusions can be drawn from experiments with other drugs used in SGLT-2i.^[91,92]^

The collective experimental evidence suggests that DAPA effectively increases the binding of acetylcholine and choline receptors by inhibiting AChE activity. This action leads to an increase in the signaling connection between synapses and a significant improvement in cognitive dysfunction in patients with AD. These findings open up a new direction for using DAPA to treat AD and bring hope to more patients.

## 5. Conclusions and prospects

This article offers a comprehensive overview of the research progress on DAPA in AD, encompassing key findings from preclinical studies to clinical trials. The abundance of in vivo and in vitro evidence indicates that DAPA exerts a beneficial impact on reducing the risk of dementia. Its mechanisms may encompass antioxidative stress, antineuroinflammation, upregulation of autophagy, antiapoptosis, AChE inhibitor activity, and protection of endothelial cells against AS and BBB damage.

Although research on SGLT-2i in AD has been increasing in recent years and continues to develop in a more comprehensive and novel theoretical direction, its clinical application still faces some challenges. Studies demonstrating that SGLT-2i can reduce the risk of cardiovascular events, slow the progression of kidney disease, and prevent multiorgan fibrosis. Nevertheless, its widespread recognition as a hypoglycemic agent and potential side effects such as urinary and reproductive infections, hypoglycemic reactions, hypovolemia, and Fournier gangrene (perineal necrotizing fasciitis) may limit its use in patients with diabetes. Moreover, its clinical utility is further restricted in the presence of renal insufficiency, particularly when the glomerular filtration rate falls below 45 mL/min per 1.73 m^2^. Furthermore, due to clinical manifestations in patients with AD often lagging decades behind pathophysiological changes, determining when to use SGLT-2i in the clinic for maximum efficacy remains unknown and requires further exploration through retrospective clinical studies. Based on the current basic experimental research on AD, the classification of AD severity is inadequate. While existing literature suggests a potential therapeutic effect of SGLT-2i on AD, its optimal efficacy at different disease stages, the ideal dosage, and duration of effectiveness remain uncertain. Given that most patients with clinically diagnosed AD are in intermediate to advanced stages, the effectiveness of SGLT-2i in this population requires further validation.

Therefore, additional scientific experiments are necessary to more precisely and comprehensively elucidate the potential and value of DAPA in AD treatment.

## Author contributions

**Funding acquisition:** Lihui Liang, Shan Hui.

**Editing:** Lihui Liang.

**Conceptualization:** Ping Chen.

**Formal analysis:** Ping Chen.

**Resources:** Ping Chen, Yuhan Dai.

**Software:** Ping Chen.

**Writing – original draft:** Ping Chen.
